# Serinol: small molecule - big impact

**DOI:** 10.1186/2191-0855-1-12

**Published:** 2011-06-13

**Authors:** Björn Andreeßen, Alexander Steinbüchel

**Affiliations:** 1 Institut für Molekulare Mikrobiologie und Biotechnologie, Westfälische Wilhelms-Universität Münster, Corrensstraße 3, D-48149 Münster, Germany

**Keywords:** 2-Amino-1, 3-propanediol, Amino alcohol, Serinol

## Abstract

The amino alcohol serinol (2-amino-1,3-propanediol) has become a common intermediate for several chemical processes. Since the 1940s serinol was used as precursor for synthesis of synthetic antibiotics (chloramphenicol). In the last years, new scopes of applications were discovered. Serinol is used for X-ray contrast agents, pharmaceuticals or for chemical sphingosine/ceramide synthesis. It can either be obtained by chemical processes based on 2-nitro-1,3-propanediol, dihydroxyacetone and ammonia, dihydroxyacetone oxime or 5-amino-1,3-dioxane, or biotechnological application of amino alcohol dehydrogenases (AMDH) or transaminases. This review provides a survey of synthesis, properties and applications for serinol.

## Introduction

**Figure 1:**
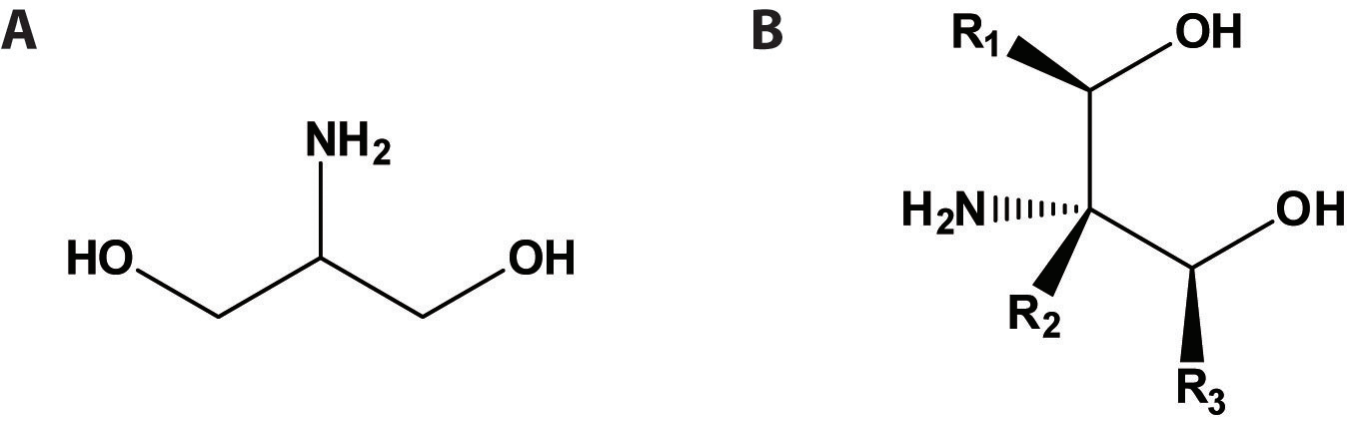
**Structural formula of serinol (2-amino-1,3-propanediol) (A) and serinol as term for the group of C-substituted commercial analogs (B)**.

2-Amino-1,3-propanediol (1,3-dihydroxy-isopropylamine, aminoglycerin or amino-trimethylenglykol) has a molecular formula of C_3_H_9_NO_2 _(Figure 1), belongs to the group of amino alcohols and is prochiral. As it is a structural analogue to the amino acid serine, the common designation is serinol. It is very stable, corrosive, hygroscopic, and dissolves very well in water. It has a molecular weight of 91.11 g/mol, melts at 52 to 56 °C, and has a boiling point of 115 to 116 °C. The term "serinol" also describes the group of C-substituted commercial analogs. As it is the case for most amino acids, serinol and its derivatives are often used intermediates in several chemical applications. In many organisms mostly eukaryotic serinol derivatives function as central second messengers. In a few prokaryotes serinol occurs as an intermediate of toxin synthesis. In this paper, we review the biological and chemical synthesis and applications for serinol and of some of its derivatives.

## Natural occurrence of serinol

Serinol occurs in sugarcane (*Saccharum officinarum*), where it can mediate the biosynthesis of the toxin helminthosporoside (2-hydroxycyclopropyl-α-D-galactopyranoside) by the pathogenic fungus *Helminthosporium sacchari *(Babczinski et al., 1978). Enzyme activity for serinol synthesis was measured with crude leaf protein extracts, pyridoxal-5-phosphate, dihydroxyacetone phosphate (D, HAP), and alanine. A *K*_m _value of 0.1 to 1 mM for serinol was determined for this enzyme. They also discovered that glutamine, glutamic, as well as aspartic acid served as amino donors for the transaminase with similar efficiencies. However, the responsible gene and protein for the transamination reaction, respectively, have not been unraveled so far.

Serinol also constitutes an intermediate in rhizobitoxine, i. e. 2-amino-4-(2-amino-3-hydropropoxy)-*trans*-but-3-enoic acid, biosynthesis by the plant pathogen *Burkholderia andropogonis *(Mitchell et al 1986) and the legume symbionts *Bradyrhizobium japonicum *and its close relative *Bradyrhizobium elkanii *(Owen et al. 1972). Rhizobitoxine is a well known inhibitor of ethylene biosynthesis. Due to this inhibition, an increased rhizobitoxine production enhances nodulation and competitiveness on *Macroptilium atropurpureum*, the purple bush-bean, or siratro (Yuhashi et al., 2000). Rhizobitoxine synthesis was most thoroughly investigated in *B. elkanii*. Tn*5 *insertion in the *rtxA *gene of *B. elkanii *caused a rhizobitoxine null mutant. The N-terminal region of RtxA has a motif homologous to several aminotransferases (Ruan and Peters 1992, Ruan et al. 1993) as the 346 N-terminal amino acids of RtxA exhibit 24% identity and 40% similarity to the aminotransferase of *Methanobacterium thermoautotrophicum *(Smith et al., 1997). Mutants with a disruption of the N-terminal part of the protein were defective in serinol accumulation (Yasuta et al 2001). The N-terminal domain of RtxA catalyzes the reaction from DHAP to serinol phosphate and further dephosphorylation to serinol (Yasuta et al. 2001). Glutamic acid, followed by alanine and aspartic acid are the preferred amino donors for this transamination reaction (Andreeßen and Steinbüchel, 2011). Insertions in the C-terminal part of the protein lead to a decrease of dihydrorhizobitoxine in *B. elkanii *USD94. The 443 C-terminal residues exhibit 41% identity and 56% similarity to the *O*-acetylhomoserine sulfhydrolase of *Leptospira meyer *(Bourhy et al., 1997). Therefore, Yasuta et al. (2001) concluded, that RtxA, exhibiting a molecular mass of 90 kDa, is a bifunctional enzyme comprising a dihydroxyacetone phosphate aminotransferase activity and a dihydrorhizobitoxine synthase activity at the same time. Dihydrorhizobitoxine is further converted to rhizobitoxine by the rhizobitoxine desaturase RtxC (Okazi et al. 2004). Introduction of the *rtxACDEFG *operon into *Agrobacterium tumefaciens *C58 resulted in serinol formation but no rhizobitoxine was synthesized (Sugawara et al. 2007).

## Natural occurrence of serinol-derivatives

**Figure 2:**
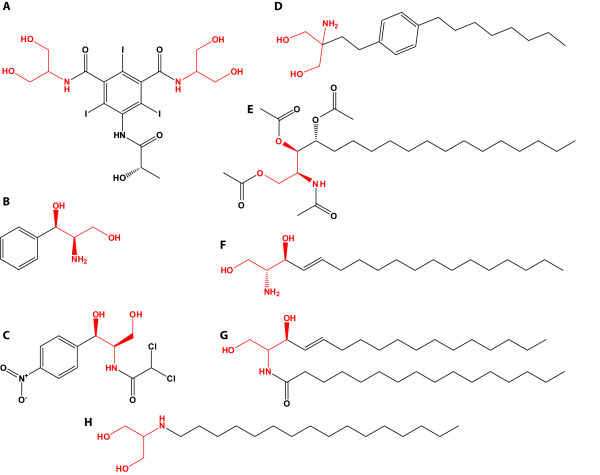
**Serinol moiety containing compounds**. **A: **iopamidol, **B: **phenylserinol **C: **chloramphenicol. **D: **fingolimod. **E: **tetra-acetyl-phytosphingosine **F: **dihydrosphingosine. **G: **ceramide. **H: **inconspicamide. The serinol units are marked in red.

Besides the already described compound serinol (2-amino-1,3-propanediol), several chiral derivatives exist. Of particular importance are the acylated serinols (sphingosines). Sphingosine (Figure 2F) and its *N*-acylated derivative, ceramide (Figure 2G), are central second messengers in eukaryotes. They are involved in regulation of cell growth, endocytosis, stress response, and apoptosis (Furuya et al. 1998, Bieberich et al. 2000, Hannun and Luberto, 2000, Uchida et al., 2003). Several serinol derivatives were obtained from marine sponges (Molinsky 2004). From *Stelletta inconspicua*, for example, the *N*-acylated serinol inconspicamide (*N*-palmitoyl-2-amino-1,3-propanediol, Figure 2H) has been extracted (Ueka et al. 2008). Spingosine and ceramide synthesis are best described in yeasts, e. g. *Saccharomyces cerevisiae*. Based on serine and palmitoyl-coenzyme A (CoA) 3-ketodihydrospingosine is condensated by a serine palmitoyltransferase and further reduced to dihydrosphingosine by a 3-ketosphinganine reductase. Ceramide is synthesized by the addition of a second palmitoyl moiety from palmitoyl-CoA, catalyzed by a ceramide synthase. Further modifications can occur by e. g. hydroxylases (Dickson and Lester, 2002). Among yeasts *Pichia ciferri *(formerly *Hansenula ciferri*) is of major industrial interest. They secrete tetra-acetyl-phytosphingosine (TAPS, Figure 2E) as the crystalline form to the medium, whereof it is easily purified (Wickerham and Stodola 1960, Casey et al. 1995, 1997, 13de Boer and van der Wildt 2001).

## Applications for serinol

In general, aminoalcohols exhibit a multitude of applications in medicine and chemical industry. Long chain α,ω-aminoalcohols serve as fungizides (Nicholas et al., 2002). Moreover, amino acid derived amino alcohols constitute important intermediates for enantiomerically pure substances (Cossy et al., 2009). Based on *N*-acetyl-1,3-amino alcohols, sphingosines for dermatological or generally pharmaceutical purposes can be synthesized (Singh et al., 2004). Since the 1940s, serinol and its commercial C-substituted analogs were a popular motif in organic compounds ([Bibr B10][Bibr B11]). Synthetic *N*-acylated serinols (*N*-palmitoyl-2-amino-1,3-propanediol) are discussed to function as anti-cancer drugs as they increase ceramide-induced (Figure 2G) apoptosis (Bieberich et al. 2000, Ueoka et al. 2008). Furthermore, the synthetic sphingosine (Figure 2F) and, since 2010 the first oral drug in multiple sclerosis treatment, fingolimod (2-amino-2-[2-(4-octylphenyl)ethyl]propane-1,3-diol, Figure 2D) distributed as Gilenya^® ^(Novartis) are synthesized from serinol (Buranachokpaisan et al., 2006). Moreover, chiral (1*R*, 2*R*) phenylserinol (Figure 2B) is a common intermediate in industrial chloramphenicol (Figure 2C) production (Darabantu et al., 1995), and aromatic L-serinol-derivatives are important intermediates for epinephrine and norepinephrine synthesis (Nakazawa et al., 1975).

Serinol is also used as an intermediate for non-ionic X-ray contrast agents like iopamidol (1-*N*,3-*N-bis*(1,3-dihydroxypropan-2-yl)-5-[(2*S*)-2-hydroxypropanamido]-2,4,6-triiodobenzene-1,3-di-carboxamide, Figure 2A), which is for example distributed as iopamiro^®^, isovue^® ^(both Bracco Diagnostics Inc.) or scanlux^® ^(Sanochemia). Iopamidol is employed as a contrast agent for angiography throughout the cardiovascular system (Villa and Paiocchi, 2003).

Furthermore, serinol constitutes a precursor for drugs dealing with pain treatment. Therefore, a straight or branched alkyl chain consisting of 12 to 22 carbon atoms is linked to the C2 atom of serinol (Michaelis et al. 2009).

## Chemical synthesis of serinol

**Figure 3:**
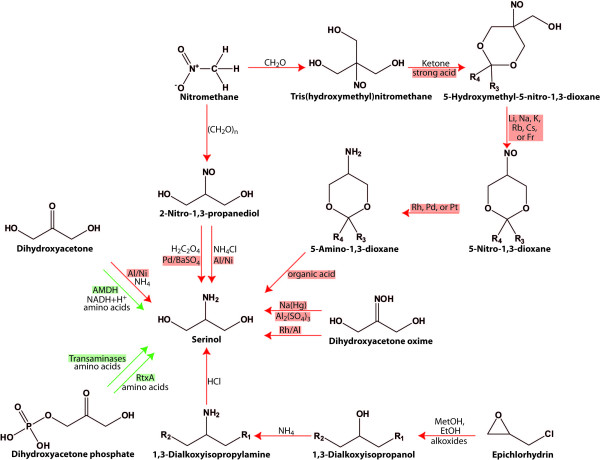
**Chemical (red arrows) and biological (green arrows) synthesis of serinol**. Catalysts are highlighted in red, enzymes are marked in green. R_1 _and R_2 _correspond to alkoxy groups, R3 and R4 represent C1-C10 alkyl, C3-C10 cycloalkyl or aryl group, or form together a C4-C10 alkylene group. AMDH: amino alcohol dehydrogenase, RtxA: dihydroxyacetone phosphate aminotransferase/dihydrorhizobitoxin synthase. Until now only the transaminase RtxA of *B. elkanii *is described. Another one is assumed for sugarcane.

Until now serinol is normally produced by chemical synthesis (Figure 3). Most of the amino alcohols or their precursors are of petro-chemical origin or need hazardous reagents during synthesis (Piloty and Ruff, 1897, Pfeiffer 1980, Thewalt et al. 1984, Felder et al. 1985, Felder et al. 1987, Fedoronko et al. 1989, Quirk et al.1989, Nardi et al. 1999, Kodali 2008). Common fossil fuel derived precursors are 2-nitro-1,3-propanediol (Pfeiffer 1980, Thewalt et al. 1984, 16 Felder et al. 1985), nitromethane (Schmidt and Wilkendorf 1919), dihydroxyacetone (DHA) (Felder et al. 1981), dihydroxyacetone oxime (Piloty and Ruff, 1897, Fedoronko et al. 1989, Nardi et al. 1999) or 5-amino-1,3-dioxane (Quirk et al.1989).

The first synthesis of serinol was reported by Piloty and Ruff (1897). They reduced dihydroxyacetone oxime with sodium amalgam in presence of aluminum sulphate. For purification serinol was converted into the corresponding hydrochloride with yields up to 15% (wt/wt) relative to the oxime starting material.

Schmidt and Wilkendorf (1919) synthesized several derivatives of 1,3-propanediol. First, *p*-formaldehyde and nitromethane condensate in presence of aqueous sodium hydroxide, then the accrued sodium salt of 2-nitro-1,3-propanediol, oxalic acid, and palladinated bariumsulfate react to serinol oxalate with yields up to 93% (wt/wt) of the theoretical value. The sodium salt of 2-nitro-1,3-propanediol was also used as raw material for serinol production by Pfeiffer (1980). Na^+^-nitropropanediol dihydrate, ammonium chloride, and raney nickel as a catalyst were solved in methanol and incubated at room temperature and 70 bar pressure. After several distillation and purification steps 75.5% (wt/wt) serinol with a purity of 99.6% were obtained. Application of palladium on carbon catalyst (5% Pd/C, 50% water) instead of raney nickel gave 74.6 to 94.5% (wt/wt) serinol recovery with about 98.7% purity (Thewalt et al. 1984). However, nitromethane as well as 2-nitro-1,3-propanediol are highly explosive. Consequently, Felder et al. (1985) used epichlorohydrin in presence of alkali with methanol or ethanol to form 1,3-dialkoxyisopropanol, which was further converted to 1,3-dialkoxy-isopropyl halide. Addition of ammonia or a primary or secondary amine formed a 1,3-dialkoxy-isopropylamine. In the last step the ether groups were separated by hydrochloric acid, yielding 80 to 91% (wt/wt) serinol with a purity of 99.8%. Furthermore, they used DHA, ammonia, and raney-nickel as a catalyst, dissolved in methanol (100 bar, 70 °C) for hydration (Felder et al. 1987). For purification, raw serinol was converted into the corresponding oxalate (yield: 87.2% wt/wt).

Quirk et al (1989) used tris(hydroxymethyl)nitromethane derived from the reaction of nitromethane and 3 moles of formaldehyde instead of DHA or 2-nitro-1,3-propanediol. Tris(hydroxymethyl)nitromethane and a ketone formed catalyzed by a strong acid (HCl or H_2_SO_4_) 5-hydroxymethyl-5-nitro-1,3-dioxane derivative. This derivative was converted into the corresponding 5-nitro-1,3-dioxane when treated with alkali. The nitro group was hydrogenated to an amino group employing rhodium, platinum or palladium catalysts. Serinol was isolated from the accrued 5-amino-1,3-dioxane in presence of a strong organic acid (Yield: 70 to 93% wt/wt). Nardi et al. (1999) used dihydroxyacetone oxime with rhodium on aluminium as catalyst, incubated it for 16 h at 70 °C and 70 bar and obtained 90% (wt/wt) of crude serinol.

However, all these manufacturing processes exhibited partial disadvantages like unsatisfactory yields, formation of dangerous by-products or poorly accessible or fossil fuel derived raw materials (Thewalt et al. 1980, Felder et al. 1987). The expense of some reactants and the required equipment led to processes unsatisfactory for industrial applications (Quirk et al. 1989). In addition, 1-amino-2,3-propandiol, which can be hardly separated from serinol, is generated during some chemical syntheses (Felder et al. 1987).

## Biotechnological synthesis of serinol

Research on biosynthesis processes depending on a biological approach was only marginal (Figure 3). Nakazawa et al. (1975) applied different aldehydes to growing cultures of *Brevibacterium helvolum*, *Candida humicola *and *Coryneacterium glycinophilum*. The highest amounts of serinol derivatives were achieved with *C. humicola *and the substrates *p-*nitrobenzaldehyde or 3,4-dinitrobenzaldehyde (8 g/l). The formation of serinol derivatives by *B. helvolum *or *C. glycinophilum *was slightly lower (*B. helvolum *and *p-*dimethylaminobenzaldehyde: 1.4 g/l, *C. glycinophilum *and *p-*nitrobenzaldehyde: 2.5 g/l).

Biotechnological production of the serinol derivatives sphingosine, dihydrosphingosine or phytosphingosine has already been established with several mutants of *Pichia ciferri*. These strains produce up to 0.8 g/l TAPS when grown under batch culture conditions (Casey et al. 1995, 1997, de Boer and van der Wildt, 2001).

Serinol can be biochemically synthesized by amino alcohol dehydrogenases (AMDH). Itoh et al. (2000) isolated a strictly NAD^+^/NADH-dependent AMDH from *Streptomyces virginiae *IFO 12827. The AMDH catalyzed the reversible dehydrogenation of serinol in presence of NAD^+ ^with a *K*_m _value of 4.0 mM to provide DHA, ammonium and NADH. The *K*_m _for the back-reaction, the reductive amination of DHA, decreased to 2.2 mM for DHA.

Our laboratory showed an artificial pathway for serinol production in recombinant *Escherichia coli*. For this, the bifunctional dihydroxyacetone phosphate aminotransferase/dihydrorhizobitoxin synthase RtxA or only its N-terminal domain (RtxA513), comprising the first reaction as described above, was heterologously expressed in *E. coli*. Up to 3.3 g/l serinol were accumulated in the supernatant by the recombinant strains, possessing whether RtxA or RtxA513, growing in presence of glycerol as sole carbon source. As no higher yields were achieved, intracellular serinol content was considered to be toxic for the cells. To lower the probable toxic effect, conversion into the corresponding acylester was intended. But an *in vitro *derivatization employing wax ester synthase/acyl-CoA:diacylglycerol acyltransferase (WS/DGAT) from *A. baylyi *ADP1 was not possible (Andreeßen and Steinbüchel, 2011).

## Conclusions

As described in this review, several applications for serinol or its derivatives are possible. Until now, large scale production of serinol is carried out via chemically processes (Piloty and Ruff, 1897, Pfeiffer 1980, Thewalt et al. 1984, Felder et al. 1985, Felder et al. 1987, Fedoronko et al. 1989, Quirk et al.1989, Nardi et al. 1999, Kodali 2008). But most of these processes are based on fossil fuel derived precursors. In times of declining oil reserves, new methods for serinol synthesis or its derivatives are needed. The knowledge about microbial alternatives, summarized by this review, offers a good starting point for further research. The fermentative production of sphingosines by *Pichia ciferri *(Casey et al. 1995, 1997, 13de Boer and van der Wildt, 2001) and serinol production from glycerol (Andreeßen and Steinbüchel, 2011) are promising examples for processes based on renewable resources.

## Competing interests

The authors declare that they have no competing interests.
